# Prediction of Immunotherapy Response and Prognostic Outcomes for Patients With Ovarian Cancer Using PANoptosis-Related Genes

**DOI:** 10.1155/humu/7108361

**Published:** 2025-10-16

**Authors:** Lei Zhang, Bo Yang, Huiting Xiao, Lu Sun, Wenting He, Ying Chen

**Affiliations:** ^1^Department of Gynecologic Oncology, Tianjin Medical University Cancer Institute & Hospital, National Clinical Research Center for Cancer, Tianjin's Clinical Research Center for Cancer, Key Laboratory of Cancer Prevention and Therapy, Tianjin 300060, China; ^2^Department of Pathology, Tianjin Medical University Cancer Institute and Hospital, Tianjin, China; ^3^Department of Gynecologic Oncology, Tianjin Medical University Cancer Institute and Hospital, Tianjin, China

**Keywords:** immunotherapy, nomogram, ovarian cancer, PANoptosis, prognosis model, tumor microenvironment

## Abstract

**Background:**

Ovarian cancer (OC) is a lethal malignancy often diagnosed at a late stage with frequent recurrence and immunotherapy resistance. PANoptosis is a novel programmed cell death regulating tumors and immunity. We constructed a prognostic model based on PANoptosis-related genes (PRGs) and evaluated its value for predicting immunotherapy response and survival in OC.

**Methods:**

PRGs linked to OC prognosis were identified from public databases, followed by using the STRING database to develop a protein–protein interaction (PPI) network. The LASSO and multivariate Cox regression analyses were used to construct a risk model, and its predictive value was verified by survival analysis, receiver operator characteristic (ROC) curve, and nomogram. Next, we analyzed the immune microenvironment by combining CIBERSORT, MCP-counter, and ssGSEA algorithms and assessed the response of patients in different risk groups to immunotherapy using TIDE with immune phenotype score (IPS) methods. GSEA was performed to evaluate the activation status of biological pathways between patients in different risk groups. Finally, we verified the expression and potential biological functions of the key genes using quantitative reverse transcription-PCR (qRT-PCR), CCK-8, scratch, and transwell assays.

**Results:**

A PANoptosis-related risk model for OC was constructed based on eight genes (*PIK3CG*, *CAMK2A*, *CD38*, *NFKB1*, *PSMA4*, *PSMA8*, *PSMB1*, and *STAT4*). The model could accurately evaluate the prognostic outcomes for OC patients, showing a high stability across different datasets. High-risk patients had lower immune cell infiltration, elevated TIDE, and reduced IPS, which suggested weaker immunotherapy responsiveness and therefore a worse prognosis. In addition, pathway analysis showed that the high-risk group was mainly enriched in tumor progression–related pathways. In vitro, *PIK3CG*, *CAMK2A*, *NFKB1*, *PSMA4*, and *PSMB1* were upregulated in OC cell lines, and knockdown of *PIK3CG* notably suppressed the proliferative, migratory, and invasive capabilities of OC cells.

**Conclusion:**

The PRG model established in this study may contribute to the assessment of immunotherapeutic response and prognosis for OC patients.

## 1. Introduction

Ovarian cancer (OC) is the third most frequent and lethal malignancy of the female reproductive system [1, 2]. Due to nonspecific symptoms, approximately 70% of OC patients are diagnosed with metastases, leading to a dismal 5-year overall survival (OS) rate of only 30% [3]. High-grade serous OC, which represents 70% of all epithelial OC cases, is recognized as the most prevalent and deadly subtype [4]. A significant challenge is that numerous patients with high-grade serous OC are detected at advanced stages, primarily due to the nonspecific symptoms of OC [5]. Optimal debulking surgery and platinum-based chemotherapy are currently the standard treatment for OC. However, up to 70% of patients eventually relapse [5]. Unlike other cancers, OC survival rates have seen minimal improvement in recent decades [6], which underscores an urgent need to explore alternative therapies to improve the outcome of OC.

Previous research focuses on the particular regulation of necroptosis, apoptosis, and pyroptosis in OC. Subsequent studies revealed extensive interactions between different cell death forms [7–9]. In 2019, Malireddi et al. [10] proposed the novel concept of “PANoptosis,” demonstrating that its activation requires coordinated involvement of pyroptosis, apoptosis, and necroptosis. PANoptosis exhibits unique biochemical characteristics. Research indicated a close correlation between PANoptosis and the onset of multiple systemic disorders, such as neurodegenerative, inflammatory, and infectious diseases [11]. Based on public databases, bioinformatics analysis screened various PANoptosis-related genes (PRGs) associated with the immunological responses of cancer patients, cancer bioprocesses, and OS. These genes were integrated to establish a PANoptosis signature to accurately evaluate the immunotherapy responsiveness and prognosis of pancreatic, gastric, prostate, and stomach cancers [9, 12–14]. It has been found that PANoptosis can improve antitumor immune responses and influence the efficacy of tumor immunotherapy [15]. Pan et al. [9] established a PAN score system in gastric cancer, where low PAN score patients showed higher immunotherapy response rates and better prognosis. However, the exact role of PANoptosis in OC remained unexplored.

Through computational analyses based on public databases, prognosis-related PRGs and predictive features related to PANoptosis for OC were discovered. We subsequently developed a PAN score to stratify patients into high- and low-risk groups based on the median risk score. The predictive utility of this model was further validated through a comprehensive assessment of prognostic differences across risk groups, including analyses of clinical parameters (e.g., grade, age, and stage), immune microenvironment characteristics, biochemical pathways, and somatic mutations. Collectively, our study established a novel PANoptosis-related risk model to predict the prognosis and immune checkpoint inhibitor (ICI) treatment response for patients with OC, providing novel insights and understanding for the clinical management of OC.

## 2. Methods

### 2.1. Data Acquisition

The gene expression profiles and clinical data for the OC patients were sourced from GEO (https://www.ncbi.nlm.nih.gov/geo/) and TCGA (https://portal.gdc.cancer.gov/) databases. TCGA-OC FPKM values were log2-transformed and converted to transcripts per kilobase million (TPM). A total of 373 samples in TCGA-OC cohort (training cohort) and 260 OC samples in the GSE32062 dataset (validation cohort) with full survival data were included. In addition, we downloaded the gene sets of five related pathways from the MSigDB database (https://www.gsea-msigdb.org/gsea/msigdb/index.jsp), including map 04217 (KEGG_NECROPTOSIS), KEGG_APOPTOSIS, REACTOME_PYROPTOSIS, REACTOME_APOPTOSIS, and HALLMARK_APOPTOSIS, and defined their combined gene set as the PANoptosis gene set.

### 2.2. Characterization and Interaction Analysis of PANoptosis Genes in OC

First, the OC somatic mutation data acquired from TCGA database were analyzed by the R package “maftools” to characterize the mutation patterns of PANoptosis genes in OC. Next, functional associations were explored through GeneMANIA (http://www.genemania.org), which integrated comprehensive genomic and proteomic data to identify functionally similar genes and predict their roles [16]. Furthermore, we built a protein–protein interaction (PPI) network based on the functionally similar genes using the STRING database (https://cn.string-db.org/) with a confidence score threshold of 0.7. The network was visualized using Cytoscape (v3.8.0) for further topological analysis [17].

### 2.3. Development of a Risk Model and Validation

PANoptosis genes closely linked to the prognosis of OC were filtered by univariate Cox regression analysis, followed by LASSO Cox regression using the R package “glmnet” [18, 19] to reduce gene number and prevent overfitting [20]. Stepwise regression analysis determined key genes for developing a PANoptosis-related gene score (PRG score), which was computed as follows: PRG score = *Σβi* × Expi. The PRG score of each patient in the training and validation cohorts was calculated, based on which the high-risk group (zscore > 0) and low-risk group (zscore < 0) were classified. After standardizing the risk scores to zscore, survival analyses for the two risk groups were conducted utilizing the R software package “survminer” [21]. We performed time-dependent receiver operator characteristic (ROC) analysis to assess 1-, 3-, and 5-year area under ROC curve (AUC) values through the “timeROC” package [22].

### 2.4. Correlation Between Immune Checkpoint Genes and PRGs

The relationship between the immune checkpoint–associated genes and eight PRGs screened by above analysis was analyzed using the “GGPUBR,” “ggplot2,” and “ggExtra” R packages [23], with statistical significance set at *p* < 0.05.

### 2.5. Analysis of Immune Microenvironment and Immunotherapy

The immune microenvironment of OC tissues was comprehensively evaluated applying multiple computational methods. CIBERSORT and MCP-counter algorithms were used to quantify the infiltration of diverse immune cell types [24]. The immune activity of each sample was further assessed using ssGSEA [25]. To predict immunotherapy response, we employed the tumor immune dysfunction and exclusion (TIDE) algorithm to evaluate T cell dysfunction and exclusion based on gene expression profiles, with higher TIDE scores indicating lower responses to immune checkpoint inhibitors (ICIs) [26]. Additionally, the immune phenotype score (IPS) was calculated using TCIA data to estimate potential responses to PD-1 and CTLA-4 blockade therapies [27, 28].

### 2.6. Pathway Analysis

We performed pathway analysis of the HALLMARK pathway gene sets acquired from the MSigDB database (https://www.gsea-msigdb.org/gsea/msigdb/) using the GSEA method [25]. HALLMARK enrichment scores calculated by ssGSEA were used to compare pathway activity among different groups.

### 2.7. Culture of Cells and Temporary Transfection

Human normal ovarian cell line IOSE-80 (YS2273C, RRID: CVCL_5546) was purchased from Yaji Bio (Shanghai, China), and OC cell line SK-OV-3 (BNCC310551, RRID: CVCL_0532) was purchased from BeNa Culture Collection (Xinyang, China). The cells were cultured in Roswell Park Memorial Institute (RPMI) 1640 medium (11875093, Gibco, Waltham, MA, United States of America) supplemented with 10% fetal bovine serum (FBS, 10099141, Gibco, United States of America), 100 *μ*g/mL streptomycin, and 100 *μ*g/mL penicillin G (15140122, Gibco, United States of America). All these cells were cultured in an incubator at 37°C and 5% CO_2_. Following the instructions, the transfection of si-*PIK3CG* into the cells was conducted applying Lipofectamine 3000 transfection reagent (L3000-001, Thermo Fisher Scientific, Waltham, MA, United States of America). The sequence of si-*PIK3CG* is as follows: si-*PIK3CG*#1: 5⁣′-GGCTAGATTATCTGAAACTGTTG-3⁣′ and si-*PIK3CG*#2: 5⁣′-TCGGTTCTTGAGATGATACTACC-3⁣′. After cell transfection, quantitative reverse transcription–polymerase chain reaction (qRT-PCR) was conducted validate the knockdown efficiency of *PIK3CG* at the mRNA level.

### 2.8. qRT-PCR

According to the instruction, total RNA was extracted from IOSE-80 and SK-OV-3 cells employing RNAiso Plus reagent (9108Q, Takara Bio, Shiga, Japan). The PrimeScript RT reagent Kit (RR037Q, Takara Bio, Japan) was used to synthesize the relevant cDNA [29]. Quantitative real-time PCR was performed using ChamQ SYBR qPCR Master Mix (Q711-02, Vazyme, Nanjing, China), with GAPDH as the internal control. Each reaction was carried out in triplicate, and gene expressions were calculated using the 2^−ΔΔCt^ method. See Table 1 for primer sequences used for qPCR.

### 2.9. Cell Counting Kit 8 (CCK-8) Determination

SK-OV-3 cells in the logarithmic growth phase were harvested and seeded into 96-well plates at a density of 3 × 10^4^ cells/mL (100 *μ*L/well, approximately 3000 cells per well). After incubation at 37°C with 5% CO_2_ for 48 h, 10 *μ*L of CCK-8 solution (CK04, Dojindo, Kumamoto, Japan) was added to each well (medium: CCK‐8 = 10 : 1), followed by incubation for 3 h. The absorbance was measured at 490 nm using a microplate reader to assess the cell viability [30].

### 2.10. Wound Healing Test

Assessment of collective cell movement in wound healing experiments was conducted using a six-well plate. Briefly, cells in the logarithmic growth phase were digested, resuspended in cell suspension, and then seeded into the six-well plate at a density of about 5 × 10^5^ cells/well. Once the cell density exceeded 90%, artificial wounds were created using a sterile 100 *μ*L pipette tip, and the healing of the “injured” monolayer was evaluated after 48 h.

### 2.11. Transwell Assay

Transwell chambers (8 *μ*m pore size; Corning, Inc., Corning, NY, United States of America) were used to assess the migration of SK-OV-3 cells. 36 hours post transfection, 200 *μ*L of SK-OV-3 cells (2 × 10^4^ cells/mL) in serum-free medium were seeded into the upper chamber, while 700 *μ*L of medium containing 10% FBS was added to the lower chamber. Nonmigrated cells were removed after incubation for 48 h at 37°C, while migrated cells were fixed by 4% paraformaldehyde and colored with 0.1% crystal violet. Cells were photographed under a microscope from six random fields and quantified by ImageJ software [31].

### 2.12. Statistical Analyses

Analysis and visualization of all statistical data were realized using R (Version 3.6.0) and GraphPad Prism (version 10.0.4). Wilcoxon's rank-sum test was used to compare the difference between continuous variables in two groups, and Spearman's method was used to assess the correlation. Log-rank test was utilized to compare survival differences between the two risk groups. For the experimental data, the one-way/two-way analysis of variance and unpaired *t* test was applied. A *p* < 0.05 was established as the threshold of statistical significance.

## 3. Result

### 3.1. Mutations and Interactions of Prognosis-Related PANoptosis Genes in OC

**Figure 1:**
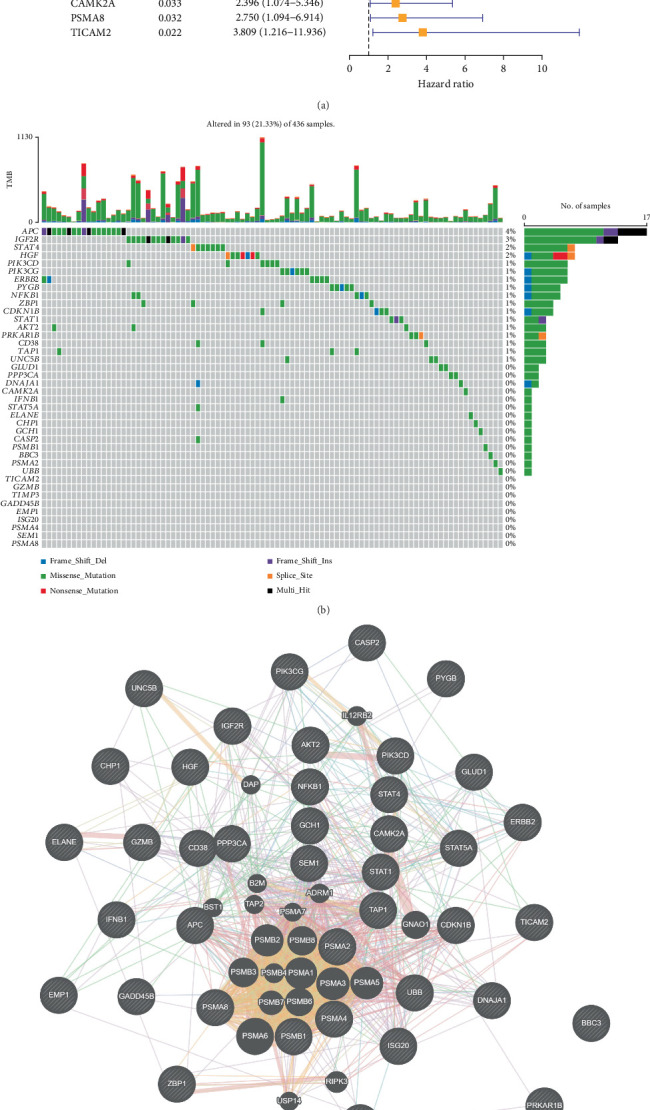
Mutation and interaction profiles of PANoptosis-related genes in OC samples. (a) Correlation of each gene with the prognosis of OC patients. (b) The mutation information of PANoptosis genes in TCGA-OC samples in a waterfall plot, with color annotations used to distinguish different mutation types. The bar at the top of the legend indicates the mutation load, while the bar on the right displays the distribution of mutation types in the gene. (c) Using the GeneMANIA website, an interaction network comprising 40 important prognostic genes was built. Each gene is represented by a node, and the various colors of the network edges indicate the possible roles of the associated genes. (d) The interaction networks of 22 genes were examined using the STRING database.

A total of 486 genes related to OC prognosis were extracted from TCGA database. Univariate Cox regression analysis screened 40 genes associated with PANoptosis (Figure 1). The somatic mutation incidence of these genes in TCGA-OC samples was analyzed and visualized using the “maftools” package. Our analysis revealed generally low mutation frequencies among the examined genes, with detectable mutations only observed in *APC* (4%), *IGF2R* (3%), *STAT4* (2%), *HGF* (2%), *PIK3CD* (1%), *PIK3CG* (1%), and *ERBB2* (1%) (Figure 1). Subsequently, the GeneMANIA website was used to identify functionally similar genes and construct an interaction network for these genes. It was found that these genes were mostly associated with the proteasome core complex and related biological activities, including the presentation of antigen processing (Figure 1). By setting the minimum required interaction score to 0.7, further analysis using the STRING database identified 22 genes (including *CD38*, *PSMA8*, *PSMA4*, *PSMA2*, *PSMB1*, *NFKB1*, *STAT4*, *ERBB2*, and *PIK3CG*) that were involved in the crosstalk of pyroptosis, necroptosis, and apoptosis (Figure 1).

### 3.2. Establishment of PANoptosis-Related Prognostic Features and Validation

**Figure 2:**
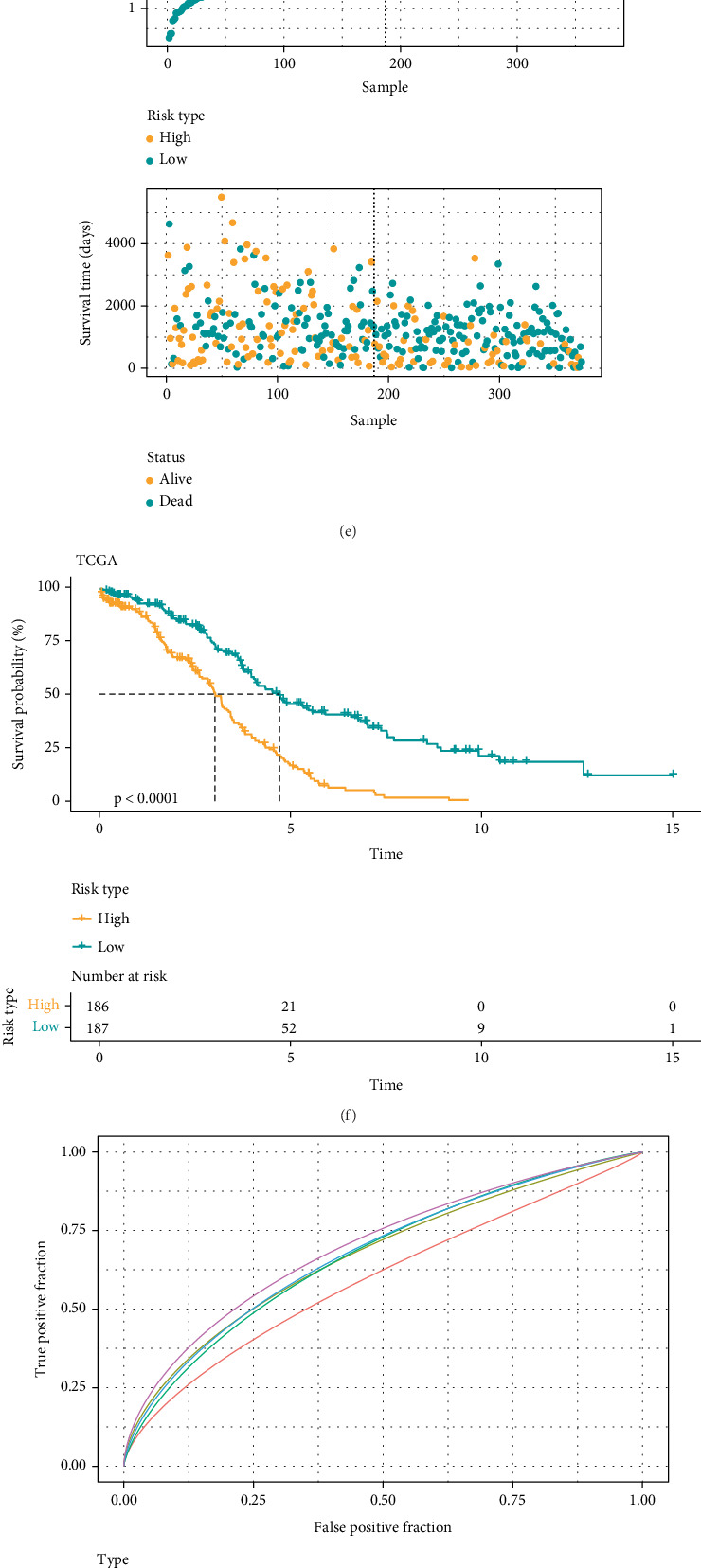
PANoptosis-associated prognostic features constructed in TCGA dataset and the GSE32062 dataset. (a) LASSO coefficient profiles of 22 prognostic PANoptosis-related genes. (b) LASSO model using cross-validation to identify the best tuned parameter (log*λ*) with log*λ* in the horizontal coordinate and mean squared error in the vertical coordinate. (c, d) Stepwise multivariate Cox regression analysis of model genes. (e) Comparison of OS in high-risk and low-risk individuals classified by their risk scores. (f) TCGA dataset was used to compare the survival of high- and low-risk individuals. (g) The model's prediction ability was evaluated by ROC curves. (h) Using the risk scores, the GSE32062 cohort was divided into high-risk and low-risk groups, and their OS was compared. (i, j) Kaplan–Meier survival curve analysis and ROC risk score analysis of the two risk groups in the GSE32062 cohort predicted the prognosis of the patients in the GSE32062 cohort. ⁣^∗^*p* < 0.05 and ⁣^∗∗∗^*p* < 0.001.

We selected characterized genes with stable prognostic value (optimal *λ* value = 0.0341) from 22 candidate PRGs based on LASSO regression and cross-validation (Figure 2a,b). The final risk model contained eight key genes, namely, *PIK3CG*, *CAMK2A*, *CD38*, *NFKB1*, *PSMA4*, *PSMA8*, *PSMB1*, and *STAT4* (Figure 2c,d). Subsequently, using the formula of PRG score = (0.573∗*PIK*3*CG* + 1.402∗*CAMK*2*A* − 0.464∗*CD*38 + 0.171∗*NFKB*1 − 0.288∗*PSMA*4 + 0.739∗*PSMA*8 + 0.436∗*PSMB*1 − 0.428∗*STAT*4), each OC patient in TCGA cohort was assigned with a risk score. Next, high- and low-risk groups were stratified by median PRG score. Notably, high-risk OC patients had shorter OS than those characterized as having a low risk (Figure 2e). Consistently, survival curve analysis showed that in comparison to patients in the high-risk group, the low-risk group had a considerably higher OS (*p* < 0.0001, Figure 2f). The PRG risk model demonstrated a high accuracy in the prognostic assessment of OC, reaching an AUC of 0.59, 0.67, 0.66, 0.67, and 0.69 for 1-, 2-, 3-, 4-, and 5-year survival prediction, respectively (Figure 2g). Subsequently, validation in the GSE32062 dataset yielded consistent results, with low-risk patients showing better outcomes (Figure 2h,i), reaching an AUC of 0.70, 0.69, 0.60, 0.56, and 0.60 for 1-, 2-, 3-, 4-, and 5-year survival prediction, respectively (Figure 2j).

### 3.3. Association Between the Prognostic and Clinical Features

**Figure 3:**
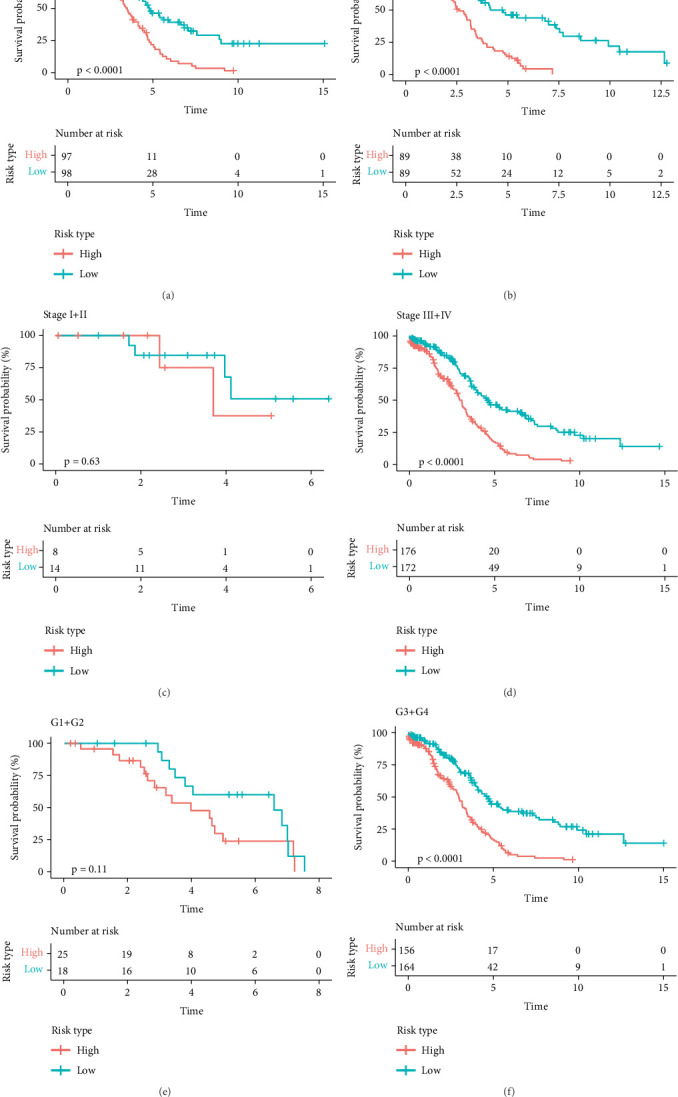
Stratified survival analysis based on clinical characteristics in TCGA cohort. OS of high- and low-risk patients as defined by the PRG score in different clinical subgroups was compared by the Kaplan–Meier curves: (a) OC patients aged ≤ 59 years, (b) OC patients aged > 59 years, (c) OC patients at Stages I–II, (d) OC patients at Stages III–IV, (e) patients with Tumor Grades G1–G2, and (f) patients with Grades G3–G4. Log-rank test assessed statistical significance.

We evaluated the association between clinical characteristics (age, stage, and grade) and risk score and found a substantial correlation between age and risk score. Specifically, older patients (> 59 years) in the high-risk group demonstrated worse prognosis compared to younger patients (≤ 59 years) (Figure 3). High-risk patients with advanced clinical stages (Stages III + IV, *p* < 0.0001) or clinical grades (Grades G3 + G4, *p* < 0.0001) showed poorer OS than those in the low-risk group (Figure 3). This suggested that our risk groupings were strongly independent and less susceptible to the interference from other clinical factors.

### 3.4. Establishment of a Clinical Nomogram for Survival Prediction in OC

**Figure 4:**
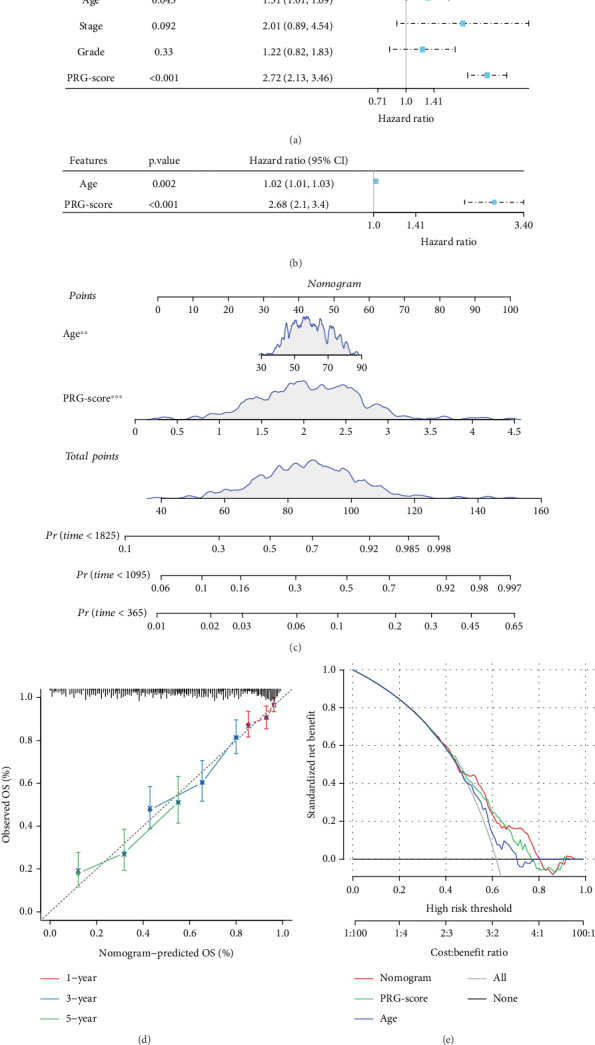
Construction process of prognostic evaluation nomogram for OC patients. (a, b) Results of univariate and multivariate Cox regression analyses. (c) The calibration curve of the 1-, 3-, and 5-year OS in OC patients was predicted using the nomogram. The nomogram's calibration and decision curve analysis findings in the (d, e) TCGA-OC queue. ns indicates *p* > 0.05; ⁣^∗∗∗^*p* < 0.001, and ⁣^∗∗^*p* < 0.01.

Univariate analysis revealed that age and PRG score were significant predictors (Figure 4a). Multivariate analysis further confirmed the significance of the two independent predictors in OC (Figure 4b). We created a nomogram (Figure 4c) integrating age and PRG score to assess the 1-, 3-, and 5-year OS for OC patients. The nomogram showed a high agreement between the predicted and actual 1-, 3-, and 5-year OS (Figure 4d). Furthermore, decision curve analysis (DCA) results further validated the superior predictive performance of the nomograms and PRG score when compared to other clinicopathologic features (Figure 4e).

### 3.5. Comparison of Tumor Microenvironment (TME) Differences Between Risk Subgroups

**Figure 5:**
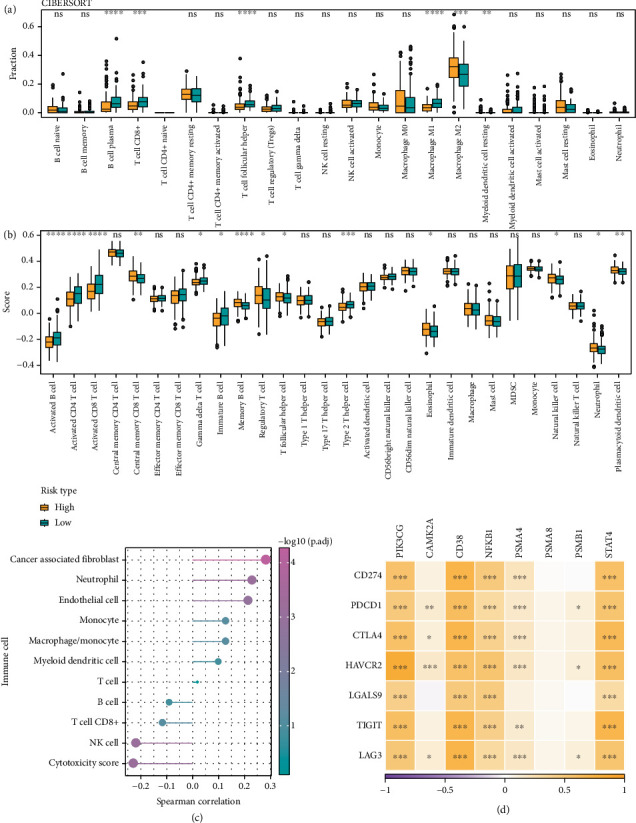
Correlation between risk score and immune infiltration in OC patients. (a) Variations in immune cell subtypes between the two risk groups. (b) Variations in infiltration of immune cells between groups at high and low risk. (c) Spearman's correlation in TCGA cohort between the PRG score and the MCP-counter immune score. (d) Immune checkpoint interaction with genes from TCGA cohort model. ⁣^∗∗∗∗^*p* < 0.0001, ⁣^∗∗∗^*p* < 0.001, ⁣^∗∗^*p* < 0.01, and ⁣^∗^*p* < 0.05; ns indicates *p* > 0.05.

We assessed immune cell infiltration in the risk subgroups in TCGA cohort to characterize and compare their immune microenvironment. According to CIBERSORT analysis, the low-risk group showed higher infiltration of T cell follicular helper, M1 macrophages, plasma B cells, and CD8^+^ T cells. The high-risk group exhibited markedly higher infiltration of M2 macrophages, indicating that these OC patients might have an immunosuppressive TME (Figure 5a). The result of ssGSEA demonstrated increased infiltration of neutrophil, eosinophil, and regulatory T cell in the high-risk group (Figure 5b). The PRG score was positively linked to cancer-associated fibroblast, neutrophil, and endothelial cell but negatively linked to cytotoxicity score, NK cells, and CD8^+^ T cells. Also, the correlation between the PRG score and MCP-counter immunity score was explored (Figure 5c). Notably, *PIK3CG*, *CD38*, *NFKB1*, and *STAT4* were the model genes that showed a positive correlation with immune checkpoint genes (Figure 5d). These data suggested that the low-risk OC group had lower infiltration of immune cells and higher immunoreactivity in their TME.

### 3.6. Characterization of Genomic Changes and Enrichment Pathways in the PANoptosis Model

**Figure 6:**
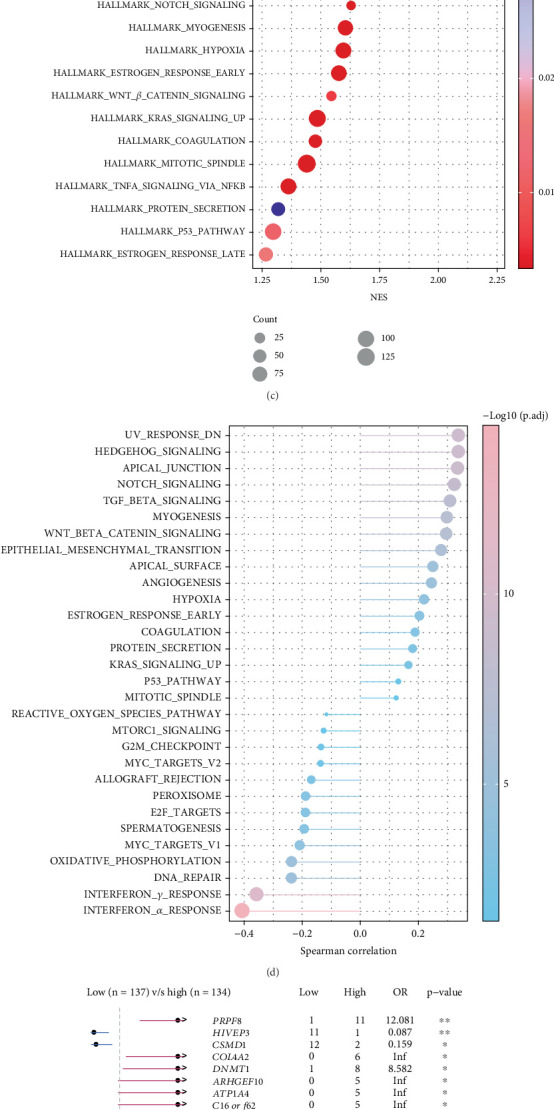
Correlation between the PANoptosis model gene and immunotherapy. (a) Variations in TIDE scores between the two risk groups. (b) Variations in IPS scores between the two risk groups. (c) The activation differences of biological pathways between the two risk groups were analyzed using GSEA. (d) PRG score and HALLMARK route score correlation. (e) Modifications in somatic mutations between groups at high and low risk. ns indicates *p* > 0.05; ⁣^∗^*p* < 0.05 and ⁣^∗∗^*p* < 0.01.

The TIDE result demonstrated notably higher TIDE scores in patients in the high-risk group (*p* < 0.05), indicating greater immune escape potential and lower treatment efficacy in this group (Figure 6a). The low-risk group responded more actively to PD-1 and CTLA-4 blockers, as evidenced by their higher IPS scores (*p* = 3.6e − 10, Figure 6b). This indicated that the low-risk group may benefit more from immunotherapy. GSEA showed that pathways associated with disease aggressiveness and progression, including “HALLMARK_ANGIOGENESIS,” “HALLMARK_HEDGEHOG_SIGNALING,” and “HALLMARK_EMT,” were significantly activated in the high-risk group (Figure 6c). Further analysis of the Spearman correlation between PRG score and HALLMARK pathway revealed that PRG score was inversely linked to the immune-related pathways (INTERFERON_ALPHA_RESPONSE, INTERFERON_GAMMA_RESPONSE) but was positively linked to several pathways (UV_RESPONSE_DN and HEDGEHOG_SIGNALING) (Figure 6d). Somatic mutation analysis detected a higher *PRPF8* mutation rate in the high-risk group (Figure 6e). Collectively, high-risk OC patients may have lower immunoreactivity in their TME and a higher possibility of immune escape during immunotherapy.

### 3.7. Expression and Functional Validation of Key Genes Screened

**Figure 7:**
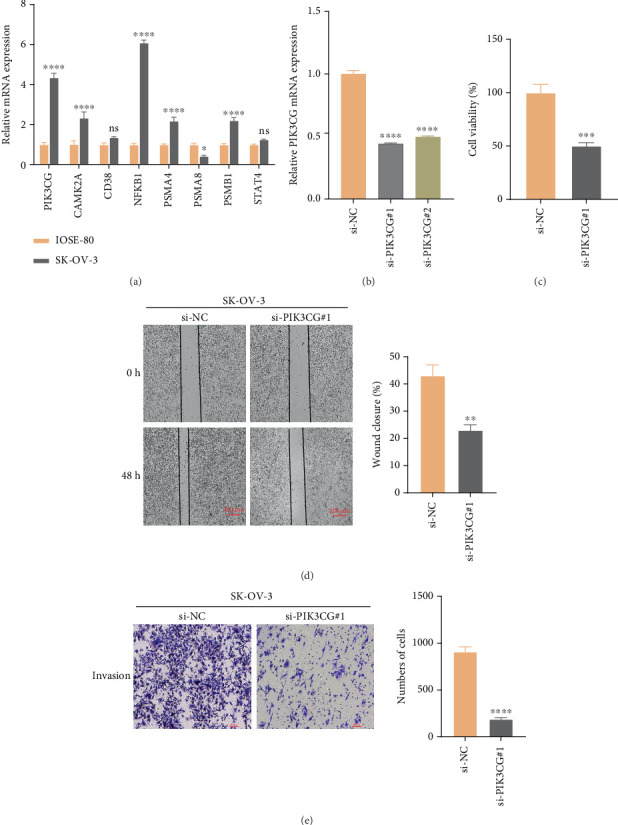
In vitro OC cell lines to validate the expression and potential biological functions of the key genes screened. (a) qPCR detected the expressions of *PIK3CG*, *CAMK2A*, *CD38*, *NFKB1*, *PSMA4*, *PSMA8*, *PSMB1*, and *STAT4* in IOSE-80 cells and SK-OV-3 cells. (b) Validation of *PIK3CG* knockdown efficiency. (c) Verification of the effect of si-*PIK3CG* on the viability of SK-OV-3 cells. (d) Representative images and statistical analysis of SK-OV-3 cells wound healing assay after *PIK3CG* knockdown. (e) Representative images of SK-OV-3 cell transwell assay and statistical analysis of invasive cell counts after *PIK3CG* knockdown. ⁣^∗^*p* < 0.05, ⁣^∗∗^*p* < 0.01, ⁣^∗∗∗^*p* < 0.001, and ⁣^∗∗∗∗^*p* < 0.0001; ns indicates *p* > 0.05.

The results of qPCR showed that *PIK3CG*, *CAMK2A*, *NFKB1*, *PSMA4*, and *PSMB1* were significantly highly expressed in SK-OV-3 cells (Figure 7). Considering that the role of *PIK3CG* in the progression of OC remained unclear, this gene was further explored. First, the qRT-PCR confirmed effective knockdown of PIK3CG by two siRNAs, with si-*PIK3CG*#1 showing a higher inhibition efficiency (Figure 7). Hence, si-*PIK3CG*#1 was used for subsequent functional experiments. The results from CCK-8 (Figure 7), scratch assay (Figure 7), and transwell assay (Figure 7) showed that *PIK3CG* knockdown (si-*PIK3CG*#1) markedly inhibited the viability, migration, and invasion of SK-OV-3 cells. These results demonstrated that the lowly expressed *PIK3CG* played a crucial role in repressing the proliferation, migratory, and invasive capabilities of OC cells.

## 4. Discussion

OC is one of the most lethal gynecological malignancies [5, 32], showing an urgent need for novel prognostic biomarkers and treatment targets. PANoptosis, which arises from the interplay between pyroptosis, apoptosis, and necrotic apoptosis, is a newly identified cell death form essential for controlling the advancement of tumors [33, 34]. This study established an eight-gene PANoptosis-based risk model (PRG score) integrating *PIK3CG*, *CAMK2A*, *CD38*, *NFKB1*, *PSMA4*, *PSMA8*, *PSMB1*, and *STAT* to help effectively predict the prognosis of OC patients. Notably, the PRG score showed high stability in both TCGA and GEO datasets. It was found that OC patients in the high-risk group had lower immune cell infiltration and poorer immunotherapy responsiveness, suggesting that PANoptosis characteristics were closely related to the tumor immune microenvironment. Additionally, in vitro experiments verified the expression of the screened genes and the proinvasive and proproliferative potential of *PIK3CG* in OC. This study was the first to systematically reveal the potential value of PANoptosis in OC prognosis and immunotherapy.

Previous studies reported a close relationship between these eight genes and tumor initiation, progression, and therapy. In particular, *PIK3CG* activation is intimately linked to tumor cell proliferation in non–small cell lung cancer, and its mutation may result in drug resistance [35]. Among the eight identified genes, *PIK3CG* showed a particularly high expression in OC cells. We also discovered that *PIK3CG* can notably enhance the migration, invasion, and proliferation of SK-OV-3 cells. *CAMK2A*, with low expression, is linked to a favorable prognosis in gliomas, but it also paradoxically correlates with reduced efficacy of immunotherapy [36]. Both hematopoietic and nonhematopoietic cells have high levels of the transmembrane glycoprotein CD38. CD38 has been found to improve the prognostic outcomes of OC patients through promoting immune infiltration and antitumor immunity in the TME [37, 38]. *NFKB1*, a key component of the NF-*κ*B pathway, can be overexpressed and regulated by norepinephrine to promote the development of OC [39]. Numerous cancers are linked to the *PSMA* family genes, which influence cell proliferation, ubiquinone breakdown, oxidative damage, and immune response signaling. High-expressed *PSMA* family genes (e.g., *PSMA2*, *PSMA3*, *PSMA4*, *PSMA6*, and *PSMA7*) in breast cancer tissues are reported to be linked to a poor OS of the cancer, but higher levels of *PSMA5* and *PSMA8* are indicative of better clinical outcomes [40]. By promoting proteasome-dependent oncoprotein degradation, *PSMB1* is regarded as a possible biomarker and a treatment target for colorectal cancer [41]. By generating signals from compounds, cytokines, growth factors, and their receptors, the *STAT* family proteins have been found to control tumor progression and treatment resistance [42]. These genes are therefore considered possible therapeutic targets that influence the metabolic reprogramming in cancers. The current model PRG score combines these genes, offering novel understanding of the mechanisms of OC and showing the potential to be used as a unique OC biomarker.

At present, the interaction between the immune environment and tumors has been widely studied as a result of the continuous breakthroughs in ICIs [43–45]. According to earlier research, PANoptosis affects the expression of immune checkpoint genes, the quantity of immune cells in the TME, and the response of these cells to immunotherapy [12, 46]. For instance, researchers discovered a strong correlation between immune cell abundance and PRG score. When colon tumors undergo PANoptosis, there is a notable increase in immunosuppressive cells (M2 macrophages) and a decrease in immune-activating cells (T cells and M1 macrophages) [12, 47]. Another study reported that in addition to effector T cells and other antitumor immune cells, the migration of Tregs is elevated in typical tumors with high PANoptosis [13]. It is commonly known that Treg cells release cytokines, prevent effector T cell growth, and facilitate immune escape in some malignancies [48]. This study evaluated the immunological infiltration landscape in OC. CIBERSORT analysis showed higher infiltration of macrophage M1, CD8^+^ T cells, plasma B cells, and T cell follicular helper in the TME of the low-risk patients. According to Raja et al. [49], OC patients with higher infiltration of CD8^+^ T cells, NK cells, and CD4^+^ T cells tend to have a better OS. The antitumor effect of a higher infiltration of M1 macrophages is consistent with our findings [50]. Interestingly, we also found that the high-risk group had higher M2 macrophage abundance, and that high M2 macrophage expression was significantly associated with immunosuppression.

In recent years, several studies constructed different types of immunotherapy prediction models for guiding the prediction of patients' responses to ICIs. For low-grade gliomas, Zou et al. created a risk score model based on glycosylation-related genes to assess patient survival; notably, their model was correlated with the immune profile, TIDE score, and treatment sensitivity, providing a new therapeutic target for gliomas [51]. In bladder cancer, Teng et al. constructed a risk score based on tumor-infiltrating immune cell (TIIC) characteristics by using multiple machine learning algorithms together with single-cell data to evaluate patients' responses to PD-L1 therapy and their prognosis [52]. In addition, in metastatic melanoma, a risk score model constructed by liquid biopsy detection of circulating soluble immune checkpoint molecules (e.g., sCTLA-4 and sCD74) has also demonstrated an accurate immune response prediction and is applicable to long-term responsive populations [53]. In contrast, this study was the first to focus on PANoptosis, a novel cell death mechanism. We constructed an eight-gene PRG score, which provided a stable and effective prognostic stratification of OC patients. The PRG score was closely linked to the TIDE and IPS scores and can reflect the state of the TME and potential immunotherapeutic response of OC patients, showing strong generalization ability and clinical adaptability. Hence, the PANoptosis signature represented an integrated scoring system that combined tumor cell death features with immune characteristics, demonstrating significant potential for identifying OC patients who may benefit from immunotherapy. However, the study also had some limitations. First, the robustness of the models may be affected by the inherent selection bias as we used retrospective datasets from public databases. Second, except for *CD38*, *NFKB1*, and *STAT4*, the underlying mechanisms of the other five genes in OC progression and the tumor immune microenvironment remained largely unknown and required further investigation. Therefore, the relationship between the eight model genes and TME during OC advancement and the underlying molecular mechanisms should be examined by in vivo and in vitro studies.

## 5. Conclusion

To conclude, this work established a PRG score, which can accurately identify high-risk OC patients and predict patients' prognostic outcomes. Correlation between high- and low-risk groups with different clinicopathological features, immune infiltration, immune response, and somatic mutations was revealed. Our study was the first to propose the potential value of PANoptosis in OC, contributing to the prognosis and clinical evaluation of targeted therapies.

## Figures and Tables

**Table 1:** qPCR primer sequences.

**Gene**	**Sequence**
*β*-Actin	F: CCCTGGAGAAGAGCTACGAG
	R: GGAAGGAAGGCTGGAAGAGT
PIK3CG	F: CCTGCAGAGCTTCTTCACCA
	R: CGTGTCCAGTACCACGTGAA
CAMK2A	F: GAGCCATTCTCACCACGATGCT
	R: TGGTGTTGGTGCTCTCTGAGGA
CD38	F: TCTTGCCCAGACTGGAGAAAGG
	R: TGGACCACATCACAGGCAGCTT
NFKB1	F: GCAGCACTACTTCTTGACCACC
	R: TCTGCTCCTGAGCATTGACGTC
PSMA4	F: CTTGTGAGCAGTTGGTTACAGCG
	R: AGCCATAGTGCTTATCCCAGCC
PSMA8	F: CCTTGATGACCATGTCTGCATGG
	R: AGTGACTGGGTCCTCAACCGTA
PSMB1	F: ACGTTTTCAACGGAGGTACT
	R: GGGCTATCCCGCGTATGAAT
STAT4	F: CAGTGAAAGCCATCTCGGAGGA
	R: TGTAGTCTCGCAGGATGTCAGC

## Data Availability

The datasets generated and/or analyzed during the current study are available in the GSE32062 repository (https://www.ncbi.nlm.nih.gov/geo/query/acc.cgi?acc=GSE32062).

## Nomenclature

OC: ovarian cancer
AUC: area under ROC curve
DCA: decision curve analysis
GEO: Gene Expression Omnibus
LASSO: least absolute shrinkage and selection operator
PRG score: PANoptosis-related gene score
OS: overall survival
ROC: receiver operating characteristic analysis
GSVA: gene set variant analysis
PRGs: PANoptosis-related genes
ssGSEA: single-sample gene set enrichment analysis
IPS: immune phenotype score
PCD: programmed cell death
TCGA: The Cancer Genome Atlas
TIDE: tumor immune dysfunction and exclusion
TME: tumor microenvironment
CTLA-4: cytotoxic T lymphocyte antigen-4
PD-1: programmed cell death protein 1
ICIs: immune checkpoint inhibitors
